# Evaluating Sentinel-2 for Lakeshore Habitat Mapping Based on Airborne Hyperspectral Data

**DOI:** 10.3390/s150922956

**Published:** 2015-09-11

**Authors:** Dimitris Stratoulias, Heiko Balzter, Olga Sykioti, András Zlinszky, Viktor R. Tóth

**Affiliations:** 1 Balaton Limnological Institute, Centre for Ecological Research, Hungarian Academy of Sciences, Klebelsberg Kuno u. 3., Tihany, H-8237, Hungary; E-Mails: zlinszky.andras@okologia.mta.hu (A.Z.); toth.viktor@okologia.mta.hu (V.R.T.); 2 University of Leicester, Centre for Landscape and Climate Research, Bennett Building, University Road, Leicester, LE1 7RH, UK; E-Mail: hb91@leicester.ac.uk; 3 National Observatory of Athens, Institute for Astronomy, Astrophysics, Space Applications and Remote Sensing, Metaxa and Vas. Pavlou Str., Penteli, Athens GR-15236, Greece; E-Mail: sykioti@noa.gr

**Keywords:** Sentinel-2, simulation, hyperspectral, spectral response function, *Phragmites*, macrophytes, lakeshore vegetation, habitat mapping

## Abstract

Monitoring of lakeshore ecosystems requires fine-scale information to account for the high biodiversity typically encountered in the land-water ecotone. Sentinel-2 is a satellite with high spatial and spectral resolution and improved revisiting frequency and is expected to have significant potential for habitat mapping and classification of complex lakeshore ecosystems. In this context, investigations of the capabilities of Sentinel-2 in regard to the spatial and spectral dimensions are needed to assess its potential and the quality of the expected output. This study presents the first simulation of the high spatial resolution (*i.e.*, 10 m and 20 m) bands of Sentinel-2 for lakeshore mapping, based on the satellite’s Spectral Response Function and hyperspectral airborne data collected over Lake Balaton, Hungary in August 2010. A comparison of supervised classifications of the simulated products is presented and the information loss from spectral aggregation and spatial upscaling in the context of lakeshore vegetation classification is discussed. We conclude that Sentinel-2 imagery has a strong potential for monitoring fine-scale habitats, such as reed beds.

## 1. Introduction

Macrophytes have complex spatial mosaic structures and, therefore, require concurrent high spatial and spectral resolution Earth Observation data. Nevertheless, the trade-off between the fixed spectral, spatial, and temporal resolution imposed by most satellite sensors currently in orbit do not allow monitoring of such complex vegetation communities. For instance Landsat-8, one of the most popular satellites for remote sensing, provides multispectral data at 30 m pixel resolution, which is too coarse for fine scale habitat mapping. Worldview-3 on the other hand, a commercial satellite with the finest spatial resolution to date, would lack the temporal resolution of Landsat as the surface coverage potential of the satellite is narrow to pass over the same area in regular intervals and acquire data with a consistent angle of view suitable for monitoring services.

Sentinel-2 is a twin-satellite mission for supporting the Copernicus program developed by the European Space Agency (ESA). The open access and free-of-charge policy adopted for the data products is anticipated to foster the use of remotely sensed data for routine environmental applications. These optical satellites offer high spatial resolution in the spectral domain 443–2190 nm for land monitoring services (including wetlands) and the first satellite, Sentinel-2A, was launched in June 2015. Enhanced specifications include the high spatial resolution, up to 10 m for selected bands, and the integration of four narrowband channels in the red-edge region [1] with the aim to provide enriched capabilities in vegetation studies [2]. One of the main advantages of Sentinel-2, which makes the satellite highly suitable for monitoring and inventorying purposes, is the combination of the wide swath and the frequent revisiting time. In this context, the sensors were designed with the scope to provide continuity to the Landsat mission.

Sentinel-2 will not only enhance the Landsat data archive but also deliver new capabilities in natural habitat mapping. For example, Hedley *et al.* [3] in a study simulating Sentinel-2 data, report an increased performance when comparing with Landsat ETM+ for mapping tropical coral reefs. Richter *et al.* [4] test the potential of Sentinel-2 for estimating Leaf Area Index (LAI) of three contrasting agricultural crops and suggest that Sentinel-2 can estimate biophysical parameters through the SAILH+PROSPECT model. Likewise, it is important to investigate the potential of the data products in anticipation of the full operations of the Sentinel-2 mission in order to assess their capabilities, examine the compatibility with satellites already in orbit, ensure continuity of image provision with similar specifications, and accelerate the operational use of the forthcoming imagery. New sensors, such as the Sentinel-2, are equipped with super-spectral resolution. It is yet to be seen whether the addition of extra bands in combination with the high spatial resolution suffices for fine-scale aquatic vegetation mapping.

This study evaluates the potential of the Sentinel-2 satellites for mapping lakeshore vegetation at the fine scale. A simulation of the high spatial resolution (*i.e.*, 10 m and 20 m) bands of Sentinel-2 on the VNIR (Visible and Near Infrared) region of the spectrum was compiled from airborne hyperspectral imagery acquired with AISA Eagle and Hawk instruments over a reed bed on the shore of Lake Balaton, Hungary. The spectral and spatial aggregations are derived separately, as well as the final simulated Sentinel-2 image. The image is classified based on the dominant land cover types encountered in the scene and reed bed subclasses. A comparison of the products with the classification of the original AISA image reveals the effect of spectral and spatial upscaling and the information lost through this process when mapping lakeshore vegetation.

## 2. Dataset and Study Area

**Figure 1:**
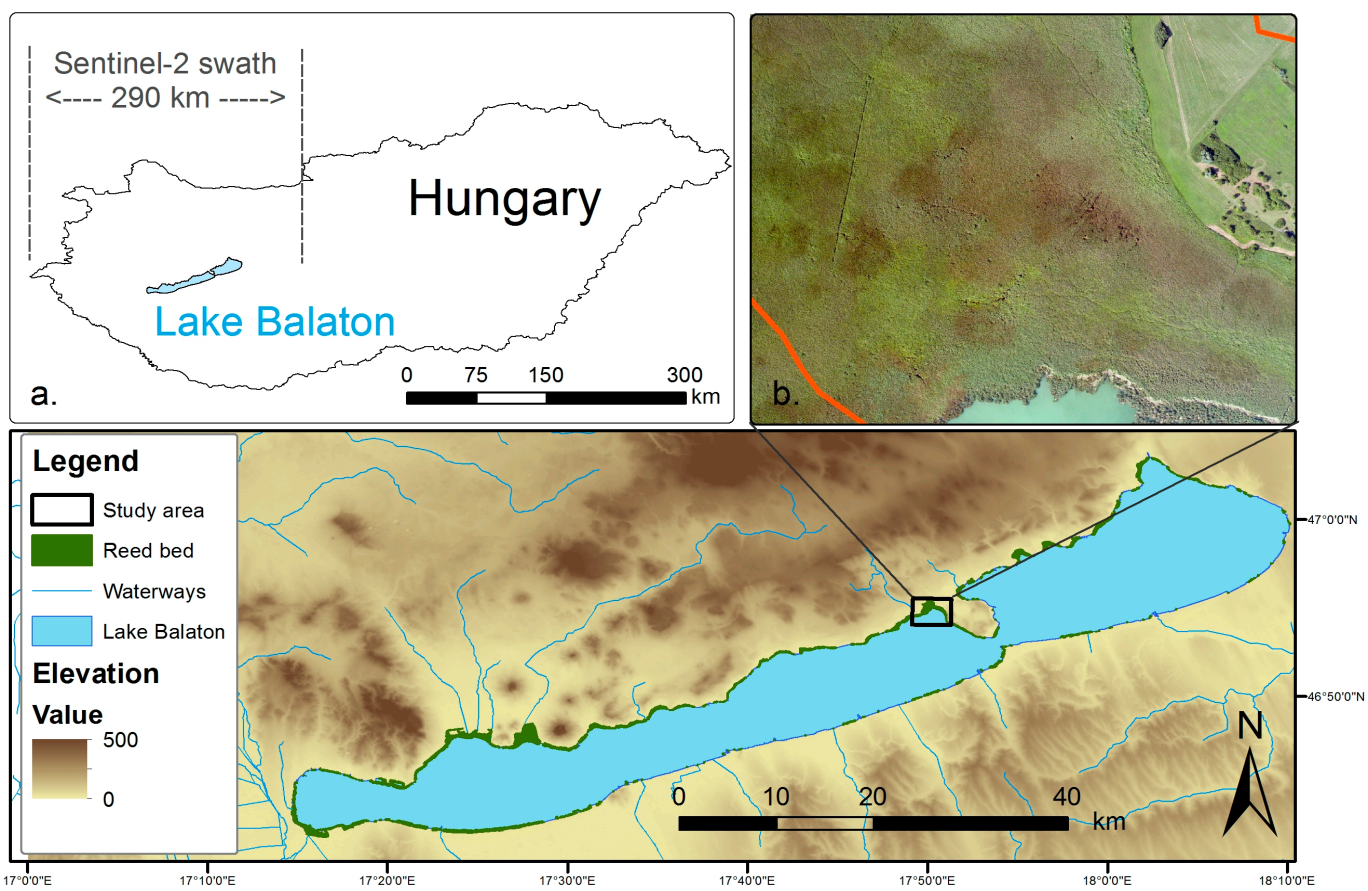
Study area in Lake Balaton, Hungary. (**a**) Position of Lake Balaton and visualization of the 290 km swath of Sentinel-2; and (**b**) true-colour RGB aerial photo (0.25 m) of the study area acquired concurrently with the hyperspectral data.

This study focuses on the east part of Bozsai Bay on the northwest part of the Tihany Peninsula of Lake Balaton, Hungary (Figure 1). The bay is a nature reserve of the Balaton Uplands National Park and quasi-undisturbed by human activity. The rare and variable vegetation encountered ranging from wetland to arid species is the result of the mild, sub-Mediterranean climate of the peninsula. It encompasses a variety of wetland plants, trees and Pannonic grasslands, however, the main ecological focus is on the dominant species *Phragmites australis* (Cav.) Trin. ex Steud, and a relatively smaller degree on *Typha angustifolia L.*, *Typha latifolia L.* and *Carex sp*. The reed bed has been suffering during the last decades from reed die-back, which is a deterioration of stability and retreat from the relatively deep water [5,6] and, therefore, apart from different species, the habitat contains vegetated patches of different stability, whereas die-back conditions occur locally at the waterward fringe of the reed bed.

An airborne hyperspectral image from a Specim AISA dual system (Spectral Imaging Ltd., Oulu, Finland) incorporating the nadir-looking sensors Eagle and Hawk was employed as in similar studies [6,7,8,9,10,11,12,13,14]. The campaign was undertaken at Lake Balaton in August 2010 [15] by the Natural Environment Research Council (NERC) Airborne Research and Survey Facility (ARSF) in the framework of the European Facility for Airborne Research (EUFAR). The sensors recorded incoming radiation cumulatively in 509 bands from 400 nm to 2450 nm with Full-Width Half-Maximum (FWHM) 2.20–2.44 and 6.32 for Eagle and Hawk, respectively, and delivered a spatial resolution of 1.5 m at 1550 m true altitude. The angle between the line of sight of the sensors and the zenith was 180° since they are both nadir looking instruments. The flying orientation of the aircraft was planned to a north-south bearing analogous to the direction of the polar orbit of the Sentinel-2 satellite (inclination 98.5°).

It is worth noting the difference in spatial coverage between the AISA and Sentinel-2 systems in Figure 1 and consider the capabilities of the latter for large-scale operational remote sensing applications; the swath width of the airborne imager is small (*i.e.*, 620 m and 990 m for Hawk and Eagle sensors, respectively) due to the low altitude of the aircraft; conversely the 290 km swath width of Sentinel-2 assures that the whole Lake Balaton is included within a maximum of two overpasses.

## 3. Methodology

The methodology is based on the spectral and spatial aggregation of the airborne hyperspectral dataset in order to synthesize an image with characteristics similar to Sentinel-2. AISA specifications are superior to Sentinel-2 specifications [16] as it inherits a higher spectral, spatial and radiometric resolution (Table 1); therefore, simulation of Sentinel-2 is realistic by aggregating the AISA data.

**Table 1:** Specifications of the Sentinel-2 sensor. The bands simulated in this study are shown in bold.

Band No.	Central Wavelength	Bandwidth	Spatial Resolution	Radiometric Resolution
1	443 nm	20 nm	60 m	12-bit
**2**	**490 nm**	**65 nm**	**10 m**	
**3**	**560 nm**	**35 nm**	**10 m**	
**4**	**665 nm**	**30 nm**	**10 m**	
**5**	**705 nm**	**15 nm**	**20 m**	
**6**	**740 nm**	**15 nm**	**20 m**	
**7**	**783 nm**	**20 nm**	**20 m**	
**8**	**842 nm**	**115 nm**	**10 m**	
**8b**	**865 nm**	**20 nm**	**20 m**	
9	945 nm	20 nm	60 m	
10	1380 nm	30 nm	60 m	
**11**	**1610 nm**	**90 nm**	**20 m**	
**12**	**2190 nm**	**180 nm**	**20 m**	

**Figure 2:**
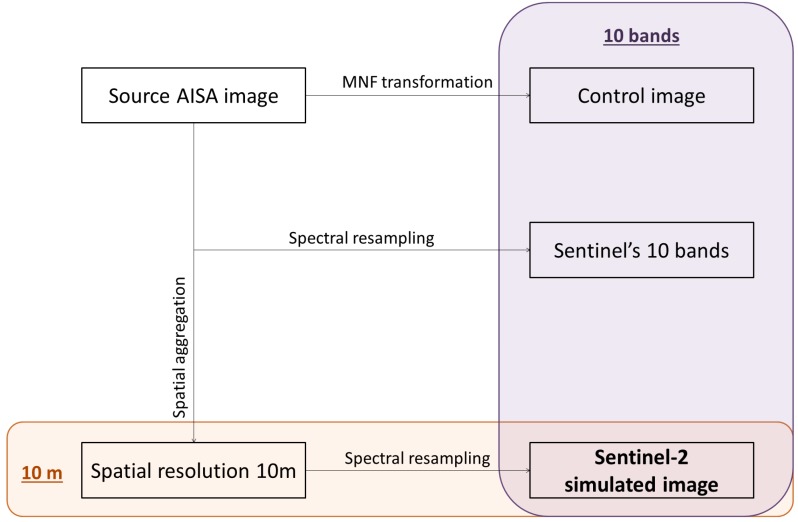
Workflow of the methodology; derivation of the four input datasets from the AISA airborne hyperspectral imagery.

The AISA dual image (Eagle and Hawk) was first pre-processed by applying radiometric normalization, atmospheric correction, and geo-registration consecutively. Cross-track illumination correction is performed using the ENvironment for Visualising Images (ENVI) 5.2 image processing software to remove sun glint resulting from the perpendicular orientation of the aircraft relative to the sun position at the time of image acquisition. Atmospheric correction was carried out with Fast Line-of-sight Atmospheric Analysis of Spectral Hypercubes (FLAASH) [17] using the mid-latitude atmospheric model without aerosol correction. Geo-registration at a 2 m pixel grid was implemented with the Airborne Processing Library software [18] using bore-sight information recorded concurrently with the imagery. Eagle and Hawk data were spectrally merged to produce a single image covering the spectrum 400–2500 nm. Thereafter the bands outside the spectrum covered cumulatively by the Sentinel-2 channels were removed in order to assure comparability between the hyperspectral and the simulated images. Subsequently the following four input products for classification were derived (Figure 2):

1. The first 10 components of the inversed Minimum Noise Fraction (MNF) transformation. This product contains the entire information from the data and is considered representative of the hyperspectral image in this set-up.

2. Spectrally resampled image. The Spectral Response Function (SRF) is convolved with the hyperspectral data for the spectral resampling. The SRF was provided by ESA-European Space Research Institute (ESRIN).

3. Spatially aggregated image. The 10 bands as of Table 1 are used. The spatial aggregation is performed individually for each narrow band according to the spatial resolution of the corresponding Sentinel-2 band (*i.e.*, 10 m or 20 m) using bilinear convolution. Bands with coarse resolution (*i.e.*, 20 m) are downscaled to 10 m and then merged with the rest of the bands. Subsequently the first 10 components of the inversed MNF transformation were derived to reduce spectral dimensionality.

4. Consecutive spatial and spectral resampling (steps 3 and 2 consecutively). This product is, in essence, the Sentinel-2 simulated image.

The four products are classified with Support Vector Machines (SVM) using the same parameters and training set. A *Phragmites*-specific classification scheme was set up as the reed bed comprises mainly of *Phragmites* co-occurring with other species. Four categories of *Phragmites* are defined according to the degree of dominance: reed dominant, co-dominant, sub-dominant, and reed die-back. Other macrophyte classes such as *Typha* and *Carex* are considered dominant at the areas where *Phragmites* is sub-dominant. Classes of trees and bare soil were included on the assumption that Sentinel-2 products will be frequently used over large areas and in more generic aquatic environment monitoring.

An assessment of relative accuracy is undertaken, indicating the loss of information when downgrading to the three different thematic maps from the source hyperspectral image, the latter considered as the “truth” data. The comparison is based on the change detection between the AISA hyperspectral classification, considered always as the control image, and each of the three simulated products which are evaluated. Emphasis is placed on the final Sentinel-2 simulated image when discussing the results. The change detection was carried out in ENVI 5.2 (Exelis Visual Information Solutions, Boulder, CO, USA) software and reports pixel values to stress the low frequency of occurrence of some classes, such as in the case of reed die-back.

## 4. Results and Discussion

**Figure 3:**
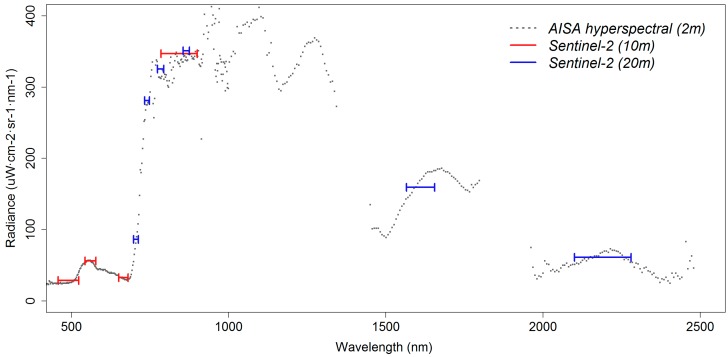
A spectral profile from a pixel of pure monospecific reed from the combined Eagle and Hawk data (grey) and the Sentinel-2 simulated image (colour).

As demonstrated in Figure 3, the Sentinel-2 bands with 10 m spatial resolution occupy the visible and near-infrared spectrum, while bands with 20 m spatial resolution are concentrated around the red-edge region and longer wavelengths encompassing the hyperspectral vegetation spectrum. From a spectral point of view, the 10 m bands are placed at wavebands containing key information for vegetation studies, as is the chlorophyll absorption characteristics. 20 m bands on the other hand have more diverse characteristics, with wide bands in the near-infrared and four narrow bands distributed in the narrow waveband of the red-edge region. It is worth noting that the red-edge bands are as narrow to include only 3–5 measurements from the corresponding hyperspectral data and, therefore, inherit a spectroscopic nature. Comparing the detail of spectra from the two sources, it becomes apparent that the near-infrared bands are averaging the hyperspectral information available over a spectral region, while in the optical and red-edge domain the waveband covered by the Sentinel-2 channels is rather constant for the case of a pure monospecific reed pixel demonstrated here.

Table 2 presents the Sentinel-2 between-band correlations for the spectrally-resampled image. The three red-edge bands are highly correlated at 0.99 which poses questions about the discriminatory capabilities of the extra bands in regards to vegetation condition and stress. Conversely, Delegido *et al*. [2] have already demonstrated the importance of Sentinel’s-2 red-edge bands for the estimation of LAI and chlorophyll content. However, it has to be taken into account that our results derive from simulated data, and the Sentinel-2 sensor might exhibit different robustness in the way radiation is recorded; hence, a real image would be necessary to judge on the inter-band correlation with certain confidence.

**Table 2:** Correlation between the spectral channels of the simulated Sentinel-2 image. The bands with correlation <0.8 are shown in bold.

Central Band	490	560	665	842	705	740	783	865	1610	2190
490 nm	1	0.99	0.91	0.80	**0.52**	**0.50**	**0.49**	**0.49**	**0.60**	**0.62**
560 nm		1	0.88	0.81	**0.58**	**0.55**	**0.55**	**0.55**	**0.61**	**0.60**
665 nm			1	0.92	**0.62**	**0.59**	**0.59**	**0.59**	**0.79**	0.85
842 nm				1	0.87	0.84	0.84	0.84	0.94	0.90
705 nm					1	0.99	0.99	0.99	0.90	**0.74**
740 nm						1	0.99	0.99	0.88	**0.71**
783 nm							1	0.99	0.89	**0.71**
865 nm								1	0.89	**0.72**
1610 nm									1	0.94
2190 nm										1

**Figure 4:**
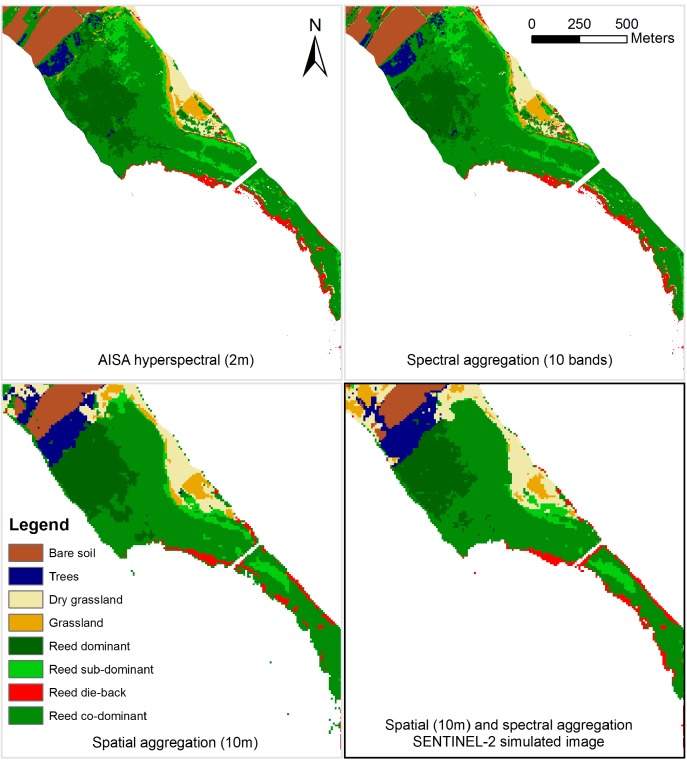
Classification of the four input products; highlighted on the lower right the classification result derived from the Sentinel-2 simulated image.

The results from the classification of the four input images are presented in Figure 4. The transitional steps of spectral or spatial aggregation indicate the level of information loss due to reduction of the spectral dimensionality and spatial upscaling from the airborne hyperspectral dataset respectively. The consecutive spatial and spectral aggregation represents the simulated Sentinel-2 image.

Classification of the spectrally-aggregated image results in a vegetation map very similar to the hyperspectral output. This indicates that the near-complete spectral information inherited by the Sentinel-2 bands contains vital information regarding vegetation targets; indeed, in such a classification set-up the superspectral characteristics of Sentinel-2 are sufficiently suitable for lakeshore vegetation mapping. However, it is worth noting that in such simulation experiments the effects of the atmospheric path, and how well it can be accounted for if a satellite product is used, cannot be simulated. While FLAASH was suitable for hyperspectral data and provides surface reflectance products based on input parameters estimated from scene information [19], the spectral resolution and bandwidth of the satellite channels will not provide adequate information in order to perform such an accurate atmospheric correction; nevertheless, the 60 m bands of Sentinel-2 will supply information on water vapor and aerosols and allow for a broad atmospheric correction based on within-scene information.

Under spatial aggregation, macrophyte-specific classes are not as well delineated as in the hyperspectral image and, therefore, capability of fine-scale mapping appears restricted. This may be attributed to the fact that spatial degradation results in larger pixels and, therefore, more classes are encountered within a single pixel. Consequently, the image is constructed from mixed pixels and the discriminatory capability between similar classes is weakened. For instance, the road network between the bare soil land parcels on the top of the image is not identifiable since the road, which is narrow compared to the pixel size, is integrated in the dominant surrounding class.

With regards to the simulated Sentinel-2 image (*i.e.*, lower right inset of Figure 4), separability between generic classes, as well as vegetation species, is maintained and moreover all classes are present. Nevertheless, misclassifications occur between macrophyte classes. Thematic consistency in generic classes such as trees, bare soil, and reed is maintained; however, the discriminatory capability is constrained when mapping classes which inherit higher inter-class spectral variability, such as macrophyte associations, fact indicated by sporadic misclassified patches. The vegetation classes of reed and trees are separated well; nevertheless, sub-dominant reed and grassland are largely being confused in the Sentinel-2 simulated image, as a result of the nature of sub-dominant reed, which most probably includes grassland at the terrestrial part of the reed bed. This suggests that similar classes, such as the aforementioned, are not distinguished as satisfactorily as in the case of the control image.

Reed die-back is classified correctly as slivers at the waterward edge of the reed bed from the Sentinel-2 data at the 10 m resolution and a complete lakeshore vegetation status seems to be feasible based on the simulated data, which would be much needed from a conservation and management perspective. Vegetation-specific applications based on Sentinel-2 imagery are further supported by the fact that mapping of vegetation vigor requires sound atmospheric correction methods, for which Sentinel-2 has dedicated bands.

**Table 3:** Assessment of relative accuracy between the classification product of the AISA hyperspectral image (control image) and the three simulated products. All values are in number of pixels.

AISA Hyperspectral Classification										
	Bare Soil	Trees	Dry Grassland	Grassland	Reed Dominant	Reed Sub-Dominant	Reed Die-Back	Reed co-Dominant	Row Total	Class Total
Sentinel-2 simulated classification										
Unclassified	3	-	10	1	-	7	497	23	541	1,266,729
Bare soil	**16,918**	17	183	9	42	21	340	721	18,251	18,290
Trees	3208	**10,344**	273	385	29,063	1744	395	9433	54,845	54,985
Dry grassland	6609	1239	**9055**	5244	4343	5105	973	12,717	45,285	46,450
Grassland	9375	108	1265	**3874**	1979	8392	65	4095	29,153	29,165
Reed dominant	-	53	-	1	**22,013**	42	-	7208	29,317	29,330
Reed sub-dominant	197	33	1382	53	333	**3309**	290	6181	11,778	11,800
Reed die-back	422	74	267	7	84	463	**4224**	5778	11,319	15,695
Reed co-dominant	908	1021	1306	121	11,192	10,877	3830	**79,177**	108,432	112,755
Class total	37,640	12,889	13,741	9695	69,049	29,960	10,614	125,333	-	-
Class Changes	20,722	2545	4686	5821	47,036	26,651	6390	46,156	-	-
Image Difference	**-19,350**	**42,096**	**32,709**	**19,470**	**-39,719**	**-18,160**	**5081**	**-12,578**	**-**	**-**
Spectral resampled (10 Sentinel bands)										
Unclassified	-	-	-	-	-	4	-	129	133	1,278,528
Bare soil	**36,004**	-	613	-	-	334	-	521	37,472	37,474
Trees	2	**5867**	1	-	805	1069	13	-	7757	7757
Dry grassland	531	-	**9240**	621	-	1049	40	236	11,717	11,717
Grassland	131	-	1439	**5662**	2	308	265	4	7811	7811
Reed dominant	25	6394	7	17	**51,821**	5281	1815	2	65,362	65,362
Reed sub-dominant	174	574	1316	1505	13,784	**109,904**	6924	1644	135,825	135,826
Reed die-back	-	54	75	1888	2636	4997	**20,898**	15	30,563	30,563
Reed co-dominant	773	-	1050	2	1	2387	5	**8067**	12,285	12,583
Class total	37,640	12,889	13,741	9695	69,049	125,333	29,960	10,618	-	-
Class Changes	1636	7022	4501	4033	17,228	15,429	9062	2551	-	-
Image Difference	**-166**	**-5132**	**-2024**	**-1884**	**-3687**	**10,493**	**603**	**1965**	**-**	**-**
Spatial aggregation (10 m)										
Unclassified	261	13	26	4	5	307	5	198	819	1,263,059
Bare soil	**21,856**	21	327	22	98	1196	41	549	24,110	24,195
Trees	2424	**11,149**	107	375	30,578	10,948	2493	138	58,212	58,415
Dry grassland	6832	251	**9350**	3948	1831	7453	1480	850	31,995	32,785
Grassland	25	16	603	**3929**	596	592	2055	13	7829	7835
Reed dominant	-	199	11	4	**25,170**	9443	69	-	34,896	34,950
Reed sub-dominant	5500	1232	2093	1190	10,533	**86,738**	18,295	4964	130,545	140,765
Reed die-back	51	-	987	198	129	5313	**4573**	176	11,427	11,475
Reed co-dominant	691	8	237	25	109	3343	949	**3726**	9088	11,720
Class total	37,640	12,889	13,741	9695	69,049	125,333	29,960	10,614	-	-
Class Changes	15,784	1740	4391	5766	43,879	38,595	25,387	6888	-	-
Image Difference	**-13,445**	**45,526**	**19,044**	**-1860**	**-34,099**	**15,432**	**-18,485**	**1106**	**-**	**-**

Table 3 presents the relative accuracy of the three simulated products based on the control image, as estimated by change detection between the two products for each instance. For the case of the simulated Sentinel-2 product, upon which the main focus is placed, it is apparent that reed classes, with the exception of reed die-back, are converted to other generic categories. Reed die-back is overestimated due to the small size of the patches, which forces the spatial aggregation of the pixels to assign the reed die-back class to larger pixels. The main misclassification is for the case of trees, due to the homogenization around the patches where individual trees or group or trees are actually found. A comparison of the three tables indicates that the misclassifications for the spectral aggregation are considerably less than the spatial aggregation and the Sentinel-2 product, a fact which indicates that the process of spatial upscaling is the main factor fostering the alteration of the classification outcome. Another observation that can be made apparent is the fact that for crisp classes (*i.e.*, bare soil, trees, dry grassland, and reed dominant) the misclassifications of the Sentinel-2 and the spatial aggregated products are similar; however, for classes with softer spectral boundaries (*i.e.*, grassland, reed sub-dominant, reed-codominant, and reed die-back) the image difference for these two products is reversed, indicating that the misclassifications of Sentinel-2 are not solely attributed to the spatial resampling, but probably to the consecutive execution of spatial and spectral resampling.

Overall, Sentinel-2 performs satisfactorily in classifying wetland ecosystems with high discrimination complexity. The 10 m spatial resolution allows for detecting fragmented patches at the edge of the reed bed, which is the main manifestation of die-back conditions. Confusion between macrophyte classes and associations is low at the individual spectral aggregation and rather high at the spatial resolution, which indicates that mapping of such classes is feasible with the spectral characteristics of Sentinel-2, but at a finer pixel resolution than that of 10 m.

## 5. Conclusions

Sentinel-2 is a twin satellite mission with enhanced spectral and spatial capabilities anticipated to provide further potential in habitat monitoring and classification of environmentally complex areas. In the framework of the recent launch of the first satellite, simulated data are needed to assess the quality of the expected product and fruitfully exploit the imagery.

In this study, a classification from simulated Sentinel-2 imagery was presented, synthesized from hyperspectral airborne data over a nature reserve site on Lake Balaton in order to assess the suitability of the imagery for fine-scale lakeshore vegetation mapping. An SVM supervised classification of a Sentinel-2 simulated image, in comparison to the MNF transformation of the hyperspectral source image, shows that the thematic consistency of generic classes such as trees, bare soil, and reed is maintained; however, when mapping classes inheriting higher inter-class spectral variability (e.g., macrophyte associations) the discrimination capability is constrained. The information is well-preserved in spectral resampling, while spatial upscaling introduces clumping of classes and mixed pixels. This study adds to the already documented high potential of Sentinel-2 for demanding Earth Observational needs [20]. Further research on the usefulness of the introduced narrow-width red-edge bands is required to investigate to what degree they can assist in stress mapping and biophysical parameters estimation.

The very large swath of Sentinel-2 (290 km) provides the opportunity of large scale main categorical mapping of large wetlands and complete lake systems, a foreseen product with high value for the conservation and management of such areas [21]. It is foreseeable that the upcoming Sentinel-2 data will enable users to derive frequent products of aquatic vegetation with wide area coverage, fine spatial resolution and thematic consistency. Moreover, synergistic use of optical data with other Earth Observation sources which have demonstrated capabilities in aquatic mapping, such as Synthetic Aperture Radar (SAR) [22] or Airborne Laser Scanning (ALS) [23] will enhance the vegetation-related information; for instance, SAR provides data associated with the inundation level, biomass, and soil moisture, while ALS relates to plant height, both cases complimentary to optical sensors’ information. In an operational context, for example, Sentinel-1, the respective SAR satellite of Sentinel-2, is already streaming free-access data and a more accurate representation of the aquatic vegetation condition could be formed when integrating the two Sentinel sources.
